# Investigation of Well-Defined Pinholes in TiO_2_ Electron Selective Layers Used in Planar Heterojunction Perovskite Solar Cells

**DOI:** 10.3390/nano10010181

**Published:** 2020-01-20

**Authors:** Muhammad Talha Masood, Syeda Qudsia, Mahboubeh Hadadian, Christian Weinberger, Mathias Nyman, Christian Ahläng, Staffan Dahlström, Maning Liu, Paola Vivo, Ronald Österbacka, Jan-Henrik Smått

**Affiliations:** 1 Laboratory of Molecular Science and Engineering, Åbo Akademi University, Porthansgatan 3-5, 20500 Turku, Finland or squdsia@abo.fi (S.Q.); mhadadia@abo.fi (M.H.); christian.weinberger@upb.de (C.W.); 2 Department of Materials Engineering, School of Chemical & Materials Engineering, National University of Science & Technology (NUST), Sector H-12, Islamabad 44100, Pakistan; 3 Department of Chemistry—Inorganic Functional Materials, Paderborn University, 33098 Paderborn, Germany; 4 Physics, Faculty of Science and Engineering, Åbo Akademi University, Porthansgatan 3-5, 20500 Turku, Finland; mathias.nyman@abo.fi (M.N.); cahlang@abo.fi (C.A.); staffan.dahlstrom@abo.fi (S.D.); rosterba@abo.fi (R.Ö.); 5 Faculty of Engineering and Natural Sciences, Tampere University, P.O. Box 541, FI-33014 Tampere, Finland; maning.liu@tuni.fi (M.L.); paola.vivo@tuni.fi (P.V.)

**Keywords:** perovskite solar cell, electron selective layer, pinhole, mesoporous TiO_2_, evaporation-induced self-assembly, dip coating

## Abstract

The recently introduced perovskite solar cell (PSC) technology is a promising candidate for providing low-cost energy for future demands. However, one major concern with the technology can be traced back to morphological defects in the electron selective layer (ESL), which deteriorates the solar cell performance. Pinholes in the ESL may lead to an increased surface recombination rate for holes, if the perovskite absorber layer is in contact with the fluorine-doped tin oxide (FTO) substrate via the pinholes. In this work, we used sol-gel-derived mesoporous TiO_2_ thin films prepared by block co-polymer templating in combination with dip coating as a model system for investigating the effect of ESL pinholes on the photovoltaic performance of planar heterojunction PSCs. We studied TiO_2_ films with different porosities and film thicknesses, and observed that the induced pinholes only had a minor impact on the device performance. This suggests that having narrow pinholes with a diameter of about 10 nm in the ESL is in fact not detrimental for the device performance and can even, to some extent improve their performance. A probable reason for this is that the narrow pores in the ordered structure do not allow the perovskite crystals to form interconnected pathways to the underlying FTO substrate. However, for ultrathin (~20 nm) porous layers, an incomplete ESL surface coverage of the FTO layer will further deteriorate the device performance.

## 1. Introduction

Organic-inorganic lead halide perovskite solar cells (PSCs) have gained substantial attention in the last decade, and soon they are expected to be able to compete with conventional silicon-based solar cells due to their outstanding device performance. PSCs offer low-cost solar energy conversion due to the ease in fabrication and the possibility to make devices on top of glass or flexible substrates [1]. The first PSC was reported by Kojima et al. in 2009 [2] with 3.8% power conversion efficiency (PCE). Within the next seven years, a PCE above 20% was reached [3], while the current certified record efficiency is, amazingly, above 25% [4]. However, there are still some challenges to be addressed before the technology can reach market penetration, including device reproducibility [5], scalability [6], and stability [7]. 

A PSC consists of a perovskite light-absorbing layer sandwiched between an electron selective layer (ESL) and a hole selective layer (HSL). Electron-hole pairs are generated in the perovskite layer upon illumination. Electrons are selectively extracted by the ESL and transported to an external circuit via a transparent conductive substrate, such as fluorine-doped tin oxide (FTO), while holes are extracted by the HSL, most commonly lithium-doped Spiro-OMeTAD. n-Type metal oxide semiconductors, including TiO_2_, ZnO and SnO_2_ [8,9], are the most commonly used ESLs in PSCs, although conductive polymers have also been used [10,11,12]. The highest efficiency reported for TiO_2_ as the ESL reaches above 20% when using cesium containing triple-cation mixed-halide perovskite as the light absorber [3]. This involves the use of a thin mesoscopic scaffold layer consisting of TiO_2_ nanoparticles coated on top of a compact TiO_2_ layer. The perovskite is thought to be embedded in the mesoporous scaffold along with a thick capping layer on top, which isolates the TiO_2_ scaffold from the HSL [13]. Together with the perovskite in the mesoporous TiO_2_, the capping layer absorbs enough light to obtain good photocurrent. The mesoscopic scaffold plays an important role in supporting electron injection into TiO_2_ because it tends to establish a high TiO_2_/perovskite interfacial area. Without the scaffold layer, it is difficult to achieve highly efficient solar cells with TiO_2_, because the low contact area between the perovskite and the TiO_2_ results in charge build-up at the interface [8,14]. This is due to the relatively low inherent electron mobility in TiO_2_ and the misalignment of its work function with respect to the conduction band energy level of the perovskite. This further becomes an important source of hysteresis in planar heterojunction TiO_2_-based PSCs [8,9].

Morphological defects such as pinholes [15] (either in the form of larger bare patches or as porosity on the nanoscale [16]) in the ESL can result in increased surface recombination as well as charge injection barriers due to improper alignment in the energy levels between the perovskite and the ESL. Pinholes in ESLs are predominantly reported as incomplete surface coverage of the ESL film on top of the conductive FTO, which results in FTO crystals protruding through the metal oxide films creating a direct contact with the perovskite [17,18]. This degrades the device performance due to a poor hole-blocking ability or high current leakage at the ESL/perovskite interfaces [9]. These types of pinholes are formed due to poor substrate wettability or microbubble formation during the film deposition or drying stages and are most prominent in ultrathin metal oxide films. Thermally or mechanically induced cracks might also expose the underlying FTO in relatively thick ESLs [1,19]. Furthermore, the choice of deposition technique and film deposition rate are important factors that affect the surface coverage and the denseness of the produced ESL layer [16]. Atomic layer deposition (ALD) is currently considered the best deposition method for producing homogeneous and dense metal oxide thin films with well-defined morphology and crystallinity [16,20]. Whereas the atomic layer-by-layer structure buildup in ALD creates very dense metal oxide layers, the more commonly used solution-processable nanoparticle- or sol-gel-based methods typically produce layers with a lower density, as they contain some porosity at the nanoscale [16]. The increased porosity and grain boundaries in the ESLs are thought to increase the number of trap states where charge recombination can take place [21]. Nonetheless, due to expensive ALD equipment costs, sol-gel-based spray pyrolysis and spin coating are still the most popular methods for producing the compact metal oxide layers used in PSCs [22]. Moreover, some of the possible trap state defects can be alleviated by surface passivation strategies [18]. 

As the nature of the possible defect morphologies in ESLs can vary substantially (in the literature irregular and randomly occurring defect structures are often encountered), it is difficult to study their possible effects in the performance of PSCs systematically. In this study, we deliberately introduce well-defined morphological defects to model nanoscale pinholes evenly distributed throughout ~70 nm thick TiO_2_ films. The sol-gel-derived films are prepared using an inexpensive, well-controlled, and scalable dip-coating method [23] in combination with evaporation-induced self-assembly (EISA) of block co-polymers to generate an ordered pore structure in the final films with a pore size of ~10 nm in diameter [24]. Such porous films have previously been utilized in PSCs, but only in combination with an additional compact TiO_2_ layer underneath [25]. In our study, however, the porosity in the TiO_2_ films is tuned to simulate a large number of uniform pinholes in a single TiO_2_ layer. The TiO_2_ layers are characterized using grazing incidence X-ray diffraction (GI-XRD), X-ray reflectometry (XRR), field emission scanning electron microscopy (FE-SEM) and charge extraction (of injected carriers) by linearly increasing voltage in metal-insulator-semiconductor structures (MIS-CELIV) measurements, confirming that the pore system is achieved through the entire TiO_2_ layer and thus forms narrow pinholes down to the underlying FTO contact. Unexpectedly, devices based on TiO_2_ layers with high porosity still work very well. This indicates that narrow pinholes similar to the ones described by our model system do not have a significant negative impact on the device performance. This further implies that variations in the density of the TiO_2_ layer when using different deposition methods or deposition rates are less important as long as the ESL fully covers the FTO substrate.

## 2. Materials and Methods 

To prepare the ordered mesoporous TiO_2_ films, a modified protocol of the one described by Ortel et al. [26] was used. However, instead of using triblock-based co-polymers, we used the commercially available poly(butadiene(1,4 addition)-b-ethylene oxide diblock co-polymer (P2952_BdEO, MW = 13,000 g/mol, Polymer Source Inc., Dorval, QC, Canada) in our study. Two independent parameters were studied: (1) block co-polymer content (i.e., change in porosity), and (2) layer thickness (while keeping the porosity constant). 

The TiO_2_ dip coating sols were prepared accordingly: Titanium (IV) chloride (TiCl_4_ > 99%, Fluka, Seelze, Germany) was initially diluted in ethanol (EtOH, >99.5%, ALTIA Plc, Helsinki, Finland) while stirring in an ice bath to give a 1:16 molar ratio TiCl_4_:EtOH stock solution. Mixtures of EtOH, Millipore water, and P2952_BdEO were also prepared. Subsequently, the TiCl_4_ stock solution was added dropwise to the other solutions to produce dipping sols with the final molar ratios, as listed in Table 1.

Fluorine-doped tin oxide substrates (FTO, TCO22-15, Solaronix, Aubonne, Switzerland) with dimensions 4 × 2 cm^2^ were sonicated in water, acetone, and 2-propanol for 10 min each. The samples were subsequently dried in nitrogen flow and plasma-treated for 5 min before TiO_2_ film deposition. The same cleaning protocol was used when microscope glass substrates (VWR international, cut into 2.5 × 2 cm^2^ pieces) were used. The substrates were then dip-coated with the sols described in Table 1 to produce two different series (i.e., change in porosity and change in film thickness, respectively). The dip coating was performed using a withdrawal speed of 85 mm/min at a relative humidity below 20%. The substrates were then kept at the same relative humidity in the dipping chamber for at least 5–10 min before they were transferred to the oven for calcination. The films were initially kept at 80 °C for 4 h and then heated to 475 °C at a heating rate of 1 °C/min. The samples were held at 475 °C for 15 min and then allowed to naturally cool to 150 °C. 

GI-XRD was performed on TiO_2_ films coated on top of FTO substrates using a Bruker AXS D8 Discover instrument. The measurements were performed between 24° and 40° using a step size of 0.04° and a grazing incidence angle of 0.3°. The TOPAS P software (v. 4.2) was used to calculate the TiO_2_ crystallite size using the Scherrer equation [27]. The same instrument was used for XRR analysis of TiO_2_ films deposited on microscope glass slides instead of FTO substrates, as the high roughness of FTO would distort the interference patterns of the TiO_2_ layer [23]. 2θ/ω scans were performed with an increment of 0.002°. The experimental data was fitted using the LEPTOS software (v.7.03). FE-SEM was used to determine the morphology of the TiO_2_ films with different porosity using a magnification of 100 kX, electron high tension of 2.70 kV, and an aperture size of 10 μm on a Zeiss Leo Gemini 1530 instrument. MIS-CELIV measurements using poly(3-hexylthiophene) (P3HT, Sigma-Aldrich, St. Louis, MO, USA) as charge injector layer were carried out to obtain information about the interconnectivity of the porous structure. The measurement setup and sample preparation are described in the Supplementary Information.

PSCs using the porous TiO_2_ ESLs were prepared accordingly: A 1.5 × 2 cm^2^ area from one side of the 4 × 2 cm^2^ FTO substrates was selectively etched using Zn powder (Sigma Aldrich) and 4 M HCl solution in water. The etched FTO substrates were subsequently sonicated in 2% aqueous solution of Hellmanex III detergent, water, acetone and 2-propanol for 10 min each at room temperature. The deposition of ordered porous TiO_2_ was performed by dip coating followed by calcination using the protocol described above. Kapton tape was used to mask the back side and the anode region of FTO substrate to avoid film deposition in those regions. Since there was a risk that the porous TiO_2_ films might absorb water from the moist environment, the samples were immediately transferred to a nitrogen glovebox once the samples had cooled down to 150 °C after calcination. The cesium-containing triple-cation mixed-halide perovskite light absorber layer was deposited by spin coating in combination with the anti-solvent method [3,28]. The mixed ion perovskite precursor solution contains a mixture of formamidium iodide (FAI, Greatcell Solar, Queanbeyan, Australia, 1 M), methylammonium bromide (MABr, Greatcell Solar, 0.2 M), lead iodide (PbI_2_, 99.99%, TCI Europe, Zwijndrecht, Belgium, 1.1 M), and lead bromide (PbBr_2_, 99.99%, TCI Europe, 0.2 M) dissolved in a 4:1 mixture of *N*,*N*-dimethylformamide (DMF, 99.8%, Sigma-Aldrich) and dimethyl sulfoxide (DMSO, 99.9%, Sigma-Aldrich). A 1.5 M solution of cesium iodide (CsI, 99.999%, ABCR, Karlsruhe, Germany) in DMSO was added to the above solution in a 1:19 volume ratio. This triple cation perovskite solution was spin-coated using a two-step program at 1000 and 6000 rpm for 10 and 20 s, respectively. During the second step, 200 μL of chlorobenzene (99.8%, TCI Europe) was pipetted onto the spinning substrate 5 s prior to the end of the program. The color of the films turned dark orange upon addition of chlorobenzene. Upon placing the samples on a hot plate at 100 °C, they turned dark brown within 10 s. Films were then annealed at the same temperature for 30 min. After annealing, the samples were allowed to cool down to room temperature. Subsequently the HSL solution was spin-coated on top of the perovskite layer at 4000 rpm for 30 s. The HSL solution composition was based on spiro-OMeTAD (Luminescence Technology Corporation, New Taipei, Taiwan) as the main component. Spiro-OMeTAD and 4-tert-butylpyridine (Sigma-Aldrich) were dissolved in chlorobenzene. The required amount of stock solutions of lithium bis(trifluromethylsulfonyl)imide (Li-TFSI, Sigma-Aldrich) and cobalt (III) tri[bis-(trifluromethane) sulfonamide] salt (FK209 Co (III), Greatcell Solar) in acetonitrile (99.8%, Sigma-Aldrich) were added into the first solution to obtain a molar composition of 1.0:0.5:2.5 × 10^−2^:3.3:131.5:7.2 (spiro-OMeTAD:Li-TFSI:FK209 Co (III):4-tert-butylpyridine:chlorobenzene:acetonitrile). Finally, an 80-nm-thick gold layer was deposited on top of the spiro-OMeTAD layer to form the back metal contact via evaporation at 2.5 × 10^−5^ bar. An evaporation rate of 0.1 Å/s was used for the first 10 nm, after which it increased to 0.2 Å/s until 20 nm. The rate of 0.8 Å/s was then used to evaporate further up to 80 nm. The shape of the gold contacts was circular with a diameter of 0.6 cm, i.e., the contact area was 0.28 cm^2^. 

The devices were characterized by measuring current density against voltage (J-V) scans using a 2636 Series Source Meter (Keithley Instruments, Cleveland, OH, USA) under simulated AM 1.5 sunlight close to 100 mW/cm^2^ irradiance from an Oriel Class ABB solar simulator (150 W, 2″ × 2″). The devices were masked using a black metal mask with an aperture size of 0.126 cm^2^. The J-V scans were performed between −0.3 V and 1.1 V at a scan speed of 10 mV/s, both in forward and reverse sweep. Time-dependent measurements of the devices were also performed for 5 min to determine the current densities close to their respective maximum power points (MPPs) under illumination at 1 Sun. Furthermore, for UV-Vis spectroscopy and photoluminescence (PL) measurements, TiO_2_ films deposited on glass substrates were coated with perovskite using the same deposition protocol as described above. The UV-Vis measurements were performed using a Perkin Elmer Lambda 900 UV-Vis/near infrared spectrometer. The samples were scanned in the λ range 500–900 nm and the measurements were performed in the presence of standard reflectance standards. The slit size was 2 mm. PL spectra were obtained with a FLS1000 spectrofluorometer (Edinburgh Instruments, Livingston, UK). Time-resolved photoluminescence (TR-PL) decays were determined using a time-correlated single photon counting (TCSPC) apparatus equipped with a Picoharp 300 controller and a PDL 800-B driver for excitation and a Hamamatsu R3809U-50 microchannel plate photomultiplier for detection in a 90° configuration. All samples were measured using a 648 nm excitation wavelength with an excitation energy intensity of 40 µJ/cm^2^ while exciting from the perovskite film side. The PL decays were monitored at 765 nm and well fitted with a bi-exponential function $I\left(t\right)=A_{1}\cdot e^{\left(-t/\tau_{1}\right)}+A_{2}\cdot e^{\left(-t/\tau_{2}\right)}$, where *I*(*t*) is the PL intensity at time *t*, *A*_x_ is the initial amplitude of component *x* (*x* = 1 or 2), and *τ* is the exponential lifetime of component *x* [29,30].

## 3. Results and Discussion

### 3.1. Structural Properties of the TiO_2_ Thin Films

**Figure 1:**
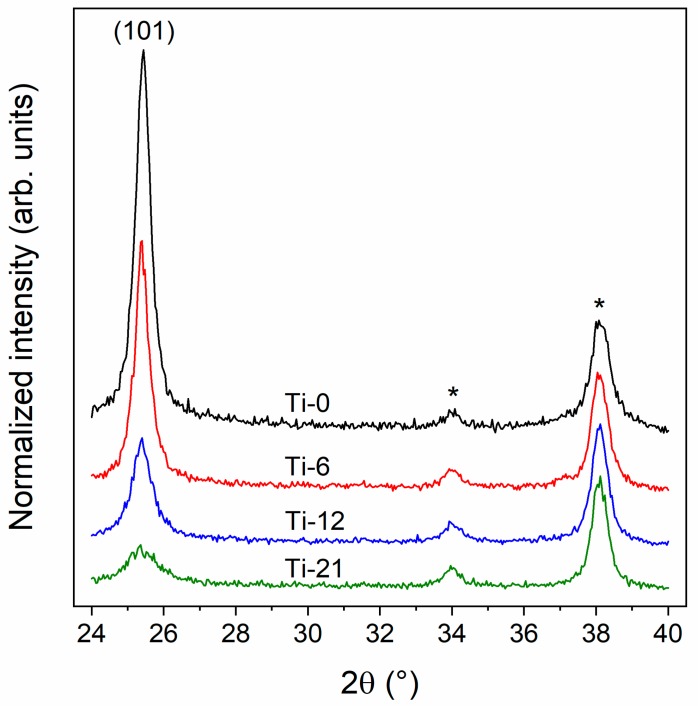
XRD diffractograms of TiO_2_ films with different block co-polymer content deposited on FTO substrates. The (101) reflection of the anatase phase is indicated in the figure. The asterisks (*) indicate reflections from the underlying FTO substrate. The diffractograms have been normalized to the intensity of the FTO reflection at 38.1° 2θ as well as offset for clarity.

The TiO_2_ thin films dip-coated on top of FTO substrates were characterized using GI-XRD to investigate the effect of block co-polymer content (i.e., film porosity) on the crystal structure. The diffractograms shown in Figure 1 reveal that all the films consist of the anatase crystal structure regardless of the amount of added block co-polymer. However, the declining peak intensity and Scherrer analysis of the full-width half maximum of the (101) reflection indicate that the anatase crystallite size decreases when higher block co-polymer amounts are used (see Table 2). The reflections at 34.0° and 38.1° 2θ originate from the underlying FTO substrate. 

TiO_2_ films dip-coated on top of planar glass substrates were studied by X-ray reflectometry (XRR) to estimate the porosity and the film thickness. Figure S1 in the Supplementary Information shows the XRR interference patterns of TiO_2_ films with different porosities. The trends in the film density and thickness as a function of block co-polymer concentration are listed in Table 2. The density of the Ti-0 reference sample is lower than the literature value for a completely crystalline anatase material (3.79 g/cm^3^). However, it is expected that nanocrystalline thin films have lower densities. With an increase in block co-polymer concentration, the density of the films decreases, meaning that the porosity originating from the block co-polymer template increases (up to 47% for the Ti-21 sample). We aimed at keeping the film thickness constant at ~75 nm regardless of the block co-polymer content to be able to directly relate the device performance to the porosity of the films. However, initial tests simply by increasing the block co-polymer amount resulted in a rapidly increasing film thickness due to the increasing viscosity of the dip coating sol. To compensate for this, a higher dilution in water and EtOH was used for higher block co-polymer to TiCl_4_ ratios (see details in Table 1). Despite this adjustment, a slight variation in film thickness was observed for the samples, ranging from 75 nm for the Ti-0 sample to 50 nm for the Ti-21 sample. Thus, in order to investigate the influence of the film thickness, we made another series based on a fixed porosity. To produce the thinnest sample, 2.5 times more solvent (EtOH and water) compared to the Ti-21 sample was used in the dipping sol (sample Ti-21-2.5), while in order to produce the thickest sample, 80% of the original solvent amount was used (Ti-21-0.8). The XRR measurements shown in Figures S1 and S2 in the Supplementary Information reveal that the film thickness of the Ti-21-2.5 sample is ~20 nm, while the Ti-21-0.8 dipping sol produces a 75-nm-thick TiO_2_ film. However, both films have a density of ~1.7 g/cm^3^, which is the same as for the original 50 nm-thick film (Ti-21-1.0). 

**Figure 2:**
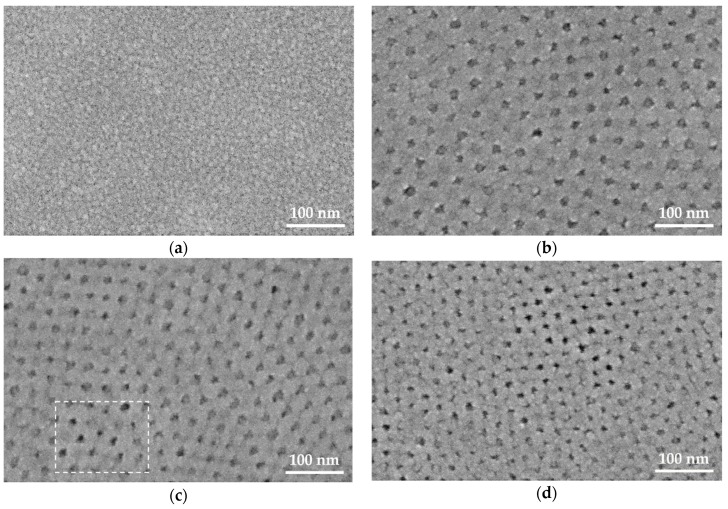
Top-view SEM images of the (**a**) Ti-0, (**b**) Ti-6, (**c**) Ti-12, and (**d**) Ti-21 thin films made on FTO substrates. The dark spherical features indicate pore openings, while the brighter areas represent the surrounding TiO_2_ wall structure (see text for further details).

**Figure 3:**
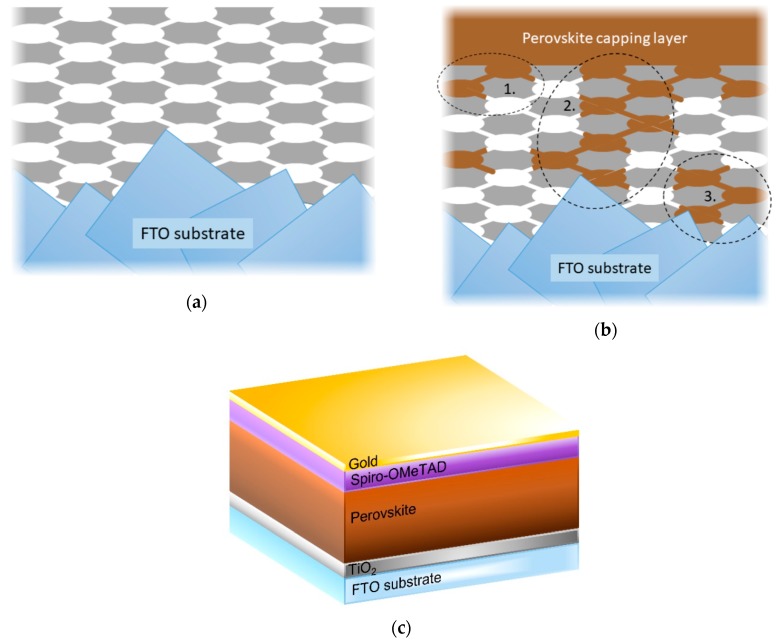
(**a**) A schematic 2-D representation of a porous TiO_2_ thin film deposited on top of a rough FTO substrate (note that the dimensions are not to scale); (**b**) suggested pore filling behavior of perovskite inside the porous TiO_2_ matrix: 1. Perovskite in contact with the perovskite capping layer, 2. direct perovskite pathway from the capping layer to FTO, and 3. isolated perovskite inside the porous matrix; (**c**) schematic illustration of the investigated device structures, where the TiO_2_ ESL is either dense or porous.

The films deposited on FTO substrates were further characterized using top-view SEM imaging, as shown in Figure 2. The Ti-0 sample shows a smooth granular surface with a grain size in the range of 10–20 nm, which is expected from the crystallite size obtained from XRD analysis. However, for the block co-polymer-templated films, pseudo-ordered mesoporous structures can be observed. The SEM images show a reduction in pore wall thickness from ~27 nm to ~15 nm with the increase in block co-polymer concentration, while the diameters of the pore openings remain roughly constant between 13 to 15 nm. At the bottom of some of the pores (highlighted in the dashed area in Figure 2c), spots with comparably darker contrast can be seen. These spots indicate the second row of pores deeper inside the TiO_2_ thin film. In the EISA process, the arrangement and interconnection of the spherical block co-polymer micellar templates determine the final pore structure [24]. In our case, it is expected that the mesopores have a body-centered-cubic (bcc) arrangement with pore shrinkage perpendicular to the substrate [26]. The ellipsoidal pores are connected via narrow channels, through which the combusted block co-polymer template escaped during the calcination process. This creates a tortuous pathway down to the underlying substrate. Based on the information obtained from XRR, XRD, and AFM, a schematic 2-D representation of the porous films can be constructed, as shown in Figure 3a.

To verify whether the underlying FTO substrate is accessible via the pores from the top, the surface recombination velocity of holes at the FTO/TiO_2_ contact was determined in model devices where the semiconducting polymer P3HT was coated on top of the different TiO_2_ films and the gold contact was evaporated on top of the P3HT. Gold forms a hole-Ohmic contact to P3HT, whereas it is well known that TiO_2_ is hole blocking. It is expected that the P3HT can fill the porous structures more easily than the perovskite, and the more P3HT that is in direct contact with FTO the higher the surface recombination would be, since FTO does not block holes [31,32,33]. The surface recombination velocity at the TiO_2_/P3HT interfaces was determined using the MIS-CELIV technique [34]. The current transients and calculated surface recombination rates for holes, S_R_, for compact and porous TiO_2_ films are shown in Figures S3 and S4 in the Supplementary Information, respectively. For the Ti-0 sample, we obtained S_R_ ≈ 10^−5^ cm/s, which is slightly higher than previously reported values [34]. We also found that S_R_ increases with increasing porosity. The Ti-6 sample has a S_R_ value one order of magnitude larger than the Ti-0 sample, and when increasing the porosity to that of the Ti-21 sample, S_R_ increases by another order of magnitude. We attribute this sharp increase to P3HT reaching all the way through the porous TiO_2_ films, down to the FTO, where holes will recombine much faster than at the TiO_2_/P3HT interface. The sharp increase in S_R_ even at low porosity would correspond to P3HT pathways forming down to the FTO, and as the porosity increases, these pathways become more accessible and/or more numerous, seen as a further increase in S_R_. This leaves us with the conclusion that the porous channels in the TiO_2_ films reaches all the way down to the FTO substrate. However, since these S_R_ measurements are limited to hole-only devices and low-mobility materials, this method cannot be used to further clarify whether these porous channels are accessible to the perovskite layer. Earlier, it has been shown that the substrate is readily accessible through thinner block co-polymer-templated TiO_2_ porous films [35,36]. Thus, we believe that the ordered mesoporous TiO_2_ films can be used as a model system for TiO_2_ ESLs with narrow and well-defined pinholes.

### 3.2. Device Performance

**Figure 4:**
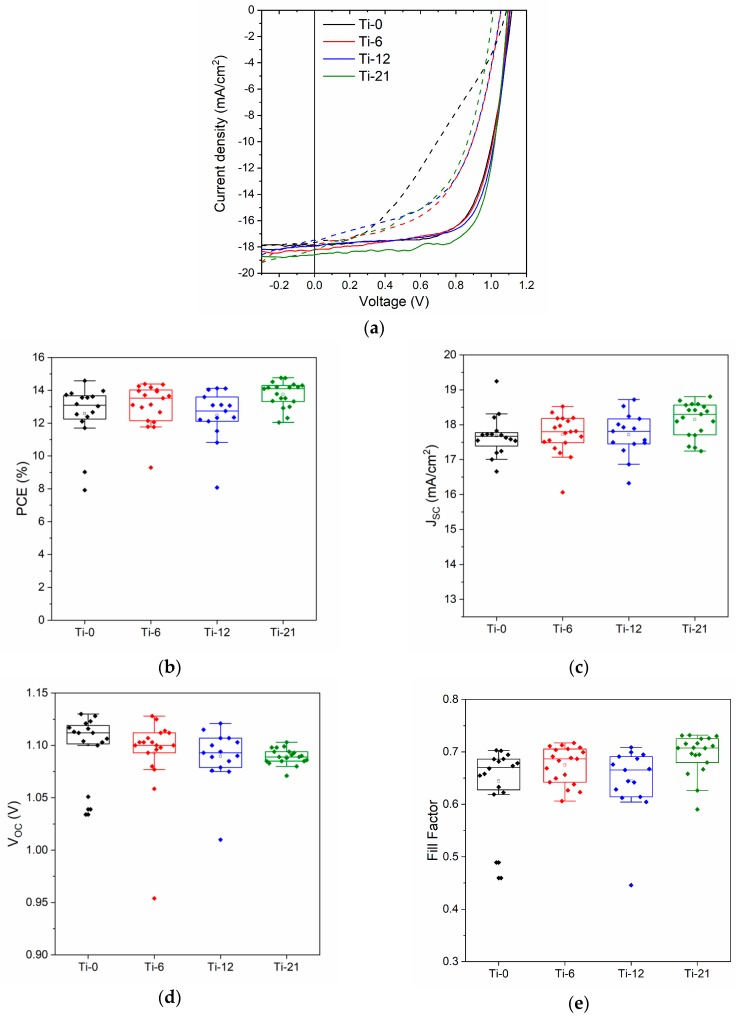
(**a**) Representative J-V curves in forward (dashed lines) and reverse (solid lines) sweep for devices with increasing porosity in the TiO_2_ layer. The solar cells were measured at a scan rate of 10 mV/s and AM 1.5 G illumination with light intensity of 100 mW/cm^2^; Changes in (**b**) device efficiencies, (**c**) *J_SC_*, (**d**) *V_OC_*, and (**e**) FF for devices measured in reverse sweep with increase in porosity in the TiO_2_ layer.

In the next step, the non-porous reference sample (Ti-0) and the ordered mesoporous TiO_2_ films with different porosities were used as ESLs in PSCs. The overall device configuration is schematically illustrated in Figure 3c. In Figure 4a, representative J-V curves of devices made with TiO_2_ ESLs with different porosities show that there are no large deviations in device performance as a function of porosity. The corresponding dark curves are shown in Figure S5 in the Supplementary Information. Furthermore, the PCE values of the devices (measured in the reverse sweep) are plotted in Figure 4b and summarized in Table 3. The devices with a dense TiO_2_ layer (Ti-0) have a mean PCE of 13.1 ± 0.7%. The reason for the lower PCE compared to previously reported values for planar PSCs based on mixed perovskites [37] is mainly attributed to the relatively large active area (~0.13 cm^2^) and substrate size, which creates a large series resistance in the device. However, the reproducibility of the device performance is still very good, which is a prerequisite for this study. 

Upon increasing the porosity in the TiO_2_ layer, the device efficiencies remain almost unchanged. The highest average efficiency (13.8 ± 0.7%) can actually be achieved for the samples with the most porous TiO_2_ layers (Ti-21). This is rather surprising, as the ordered pore structure percolates all the way through the TiO_2_ films and reaches down to the underlying FTO layer. If the perovskite were to be in direct contact with the FTO layer, one would expect shunt pathways and a considerable loss in device performance [9]. However, it seems like these narrow pore channels prevent the formation of detrimental perovskite pathways through the porous TiO_2_ to the FTO. After the high-temperature calcination of the TiO_2_ films, they were immediately transferred to the glove box in order to avoid contamination of the surface with volatile organics. Thus, the TiO_2_ pore surface is very energetic due to surface hydroxyl groups and should be readily wetted by polar solvents like the ones used in the perovskite precursor solution (DMF and DMSO). However, it is well known that the addition of the anti-solvent (chlorobenzene) brings the perovskite precursor solution into supersaturation after which a rapid perovskite crystallization commences. Our hypothesis is that the crystallization starts from the top of the porous TiO_2_ structure. The initially formed perovskite will then obstruct the pore entrances so that further precursor solution is not able to enter the pores upon solvent evaporation, but instead contributes to the perovskite capping layer. Nonetheless, the precursor solution that initially occupies the pores will be converted to perovskite upon annealing. However, as the resulting perovskite material is estimated to only occupy 15–20 vol.% in relation to the volume of the starting precursor solution (the solvents occupy the rest of the volume); this is scarcely enough to form percolating networks throughout the pore system. Instead, due to limited adhesion of the formed perovskite on the TiO_2_ surface, isolated islands of perovskite will be created inside the pore system. The suggested pore filling behavior is schematically illustrated in Figure 3b. Relating these results to the perovskite filling of nanoparticle-based mesoscopic TiO_2_ layers, the more accessible pores of those structures would allow for a considerably higher pore filling degree. In that case, an additional compact TiO_2_ layer is needed to avoid direct shunt pathways between the perovskite and FTO [23,38]. This further suggests that also the pore size could affect the pore filling degree as one would expect less pore entrance obstruction by the perovskite when the pore size is larger.

In the reverse sweep, it can be observed that the short circuit current (*J_SC_*) slightly increases with porosity (from 17.5 mA/cm^2^ to 18.5 mA/cm^2^ for the Ti-0 and Ti-21 samples, respectively). On the other hand, the open circuit voltage (*V_OC_*) marginally drops from 1.11 V to 1.09 V for the same set of samples. The opposing trends in *J_SC_* and *V_OC_* explain the rather constant PCE values regardless of porosity in the TiO_2_ ESL layer. When comparing UV-Vis absorption data of TiO_2_ films coated with perovskite (see Figure S6 in the Supplementary Information), the absorption of the perovskite in the 500–750 nm wavelength range is virtually the same for all samples with a standard deviation of ~1% at λ = 700 nm. Due to the thinness of the TiO_2_ films, we expect optical effects like increased reflectance or optical interference to be small [39,40]. Thus, the UV-Vis results further support that the perovskite located in the pore systems is proportionally low. The slight increase in J_SC_ with porosity could, however, be a result of more continuous perovskite pathways inside the pores at higher porosities, as illustrated in Figure 3b (case 1). Charges generated in isolated perovskite crystals inside the pores would normally recombine (case 3), but if more continuous pathways were to form, these charges could be extracted, and thus increase *J_SC_*. This could also explain the drop in *V_OC_*, as there will be a greater possibility that direct (shunt) pathways are formed between the perovskite capping layer and the FTO as the continuity in the perovskite pathways increase (case 2). As schematically illustrated in Figure 3b, we believe that such perovskite pathways can be formed in thinner regions of the TiO_2_ layer caused by the high surface roughness of FTO. 

To evaluate the TiO_2_ porosity-influenced electron-injection process from the perovskite conduction band (CB) to the CB of the TiO_2_ layer, steady-state photoluminescence (PL) experiments on glass/perovskite and glass/TiO_2_/perovskite samples with different porosities of the TiO_2_ layer were conducted. Figure S7 shows a clear PL quenching effect for all perovskite coated TiO_2_ films. The calculated PL quenching efficiency (PLQE), or in other words, the electron-injection yield, is increasing with decreasing porosity, suggesting that a lower porosity of the TiO_2_ layer is more favorable for an efficient electron injection process. We now turn to assess the influence of the porosity of the TiO_2_ films on the charge transfer dynamics at the perovskite/TiO_2_ interface. Figure S8 shows TR-PL decays (obtained via TCSPC measurements) of the perovskite with and without coated TiO_2_ films with different porosities together with the extracted bi-exponential fitted data of the PL decays. All PL decays of the perovskite-coated TiO_2_ films show acceleration compared to that of the pristine perovskite on glass reference, suggesting that the interfacial electron injection has occurred for all cases of perovskite/TiO_2_ films, which is also consistent with previous PL quenching data. A clear deceleration of the decay profiles is observed with increasing porosity, although the decay lifetimes for the samples with the highest porosities (Ti-12 and Ti-21) are virtually identical. Based on the reported global analysis methods [41,42,43], we attribute the first component (A_1_, τ_1_) to the trap-state-mediated recombination, while assigning the second component (A_2_, τ_2_) to the nongeminate free carrier (electron and hole) recombination and electron injection process from the CB of the excited perovskite to that of the TiO_2_ film. It is evident that the second component dominates the overall decay process, suggesting that a low porosity is more favorable for suppressing charge recombination, which in turn results in the enhanced *V_OC_* [44]. Another possibility for the change in *V_OC_* is that the increased porosity can generate more traps on the TiO_2_ structure, leading to a deepening of the TiO_2_ CB, which could intrinsically lower the *V_OC_* [30,45]. 

Furthermore, the fill factors (FF) in reverse sweep (Figure 4e) show quite similar values for all porosities in the range of 0.67–0.70. However, when comparing the device hysteresis, larger differences are observed. As seen in Figure 4a, the device based on the dense reference TiO_2_ layer (Ti-0) displays an s-shaped feature in the forward sweep, which is not observed in the reverse sweep. This results in a high hysteresis index for the device (~12%). S-shapes and high hysteresis are commonly observed for planar TiO_2_-based devices [8,9]. The s-shape is indicative of an unstabilized power output and caused by polarization of the device (most likely due to diffusion of ionic species) [46]. Upon inducing pores in the ESL, the s-shape in the forward sweep disappears, which has also been observed when shifting from planar to mesoscopic TiO_2_-based devices [46]. The hysteresis between the forward and reverse sweeps remains high (in the range of 8–10%), but the FF in the forward sweep is significantly improved upon inducing porosity in TiO_2_ films. This is also evident from the time-dependent current density measurements performed close to the maximum power point (MPP) under illumination, shown in Figure S9 in the Supplementary Information. The device based on a dense TiO_2_ film requires more time to stabilize close to the MPP than the devices based on porous TiO_2_ films. With further increase in porosity, stabilization of the current at MPP is even faster, while the magnitude of current density at the MPP is also slightly enhanced. This correlates well with the J-V curves, as an improvement in J_SC_ was observed in Figure 4a,c.

**Figure 5:**
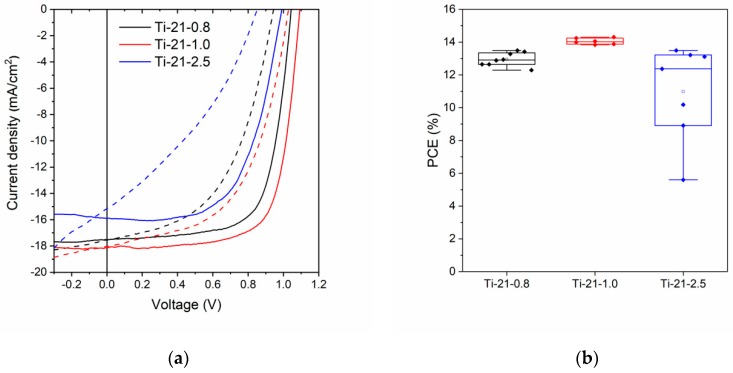
(**a**) J-V curves for representative devices based on TiO_2_ ESLs with the highest porosity with different thicknesses. Dashed lines indicate forward sweep and solid lines reverse sweep; (**b**) box chart for the efficiencies in reverse sweep.

As mentioned earlier, the thickness of the TiO_2_ films decreases slightly when more block co-polymer amounts were used, i.e., from 75 nm for the Ti-0 sample to 50 nm for the Ti-21 sample. We are aware that the device performance could be affected when the thickness of the TiO_2_ layer is altered, due to small changes in the charge transport properties or optical effects. Nonetheless, we consider the samples in porosity series to be “thick enough”, as they are all equal to or thicker than 50 nm. This is important, as it rules out the possibility that there are bare patches of FTO exposed in our devices [23]. To verify that the observed device change is an effect of the porosity rather than a change in thickness, we also made a series where the thickness of the TiO_2_ ESL was varied, while keeping the block co-polymer to TiCl_4_ ratio the same as in the Ti-21 sample. When comparing the J-V characteristics and the efficiencies in the box chart diagram in Figure 5, all thicknesses of the porous ESL display good average device performances. The devices prepared using the 75 nm-thick porous TiO_2_ films (Ti-21-0.8) perform slightly worse (PCE = 12.9%) than those prepared using the 50 nm porous TiO_2_ ESL (PCE = 14.1%). This suggests that small thickness variations can indeed also be important for optimized performance for this device structure; however, the reduced V_OC_ trend can still be observed when comparing this Ti-21 batch to the devices with less porous TiO_2_ layers in Table 3. For instance, the non-porous Ti-0 sample and the highly porous Ti-21-0.8 sample have the same film thickness (75 nm) and devices made from these samples possess roughly the same efficiency (~13%). However, when comparing the V_OC_ values for these two samples, it is clear that the V_OC_ is substantially lower for devices based on the porous TiO_2_ layer (1.05 V compared to 1.11 V). This is most likely attributed to increased charge recombination as suggested by the TR-PL data. It is noteworthy that even the devices based on a ~20 nm-thick porous TiO_2_ layer (Ti-21-2.5) display a decent average device efficiency (PCE = 11.0%). However, as seen from the scattered PCE data points in the box diagram as well as the larger standard deviations in Table 3, the thinnest sample clearly suffers from poor reproducibility (see also Figure S10 for the statistics of the photovoltaic parameters). As the porosity is roughly the same for the different thicknesses, a similar interconnectivity of the perovskite inside the pores is to be expected. Thus, the main difference between the 75 nm (Ti-21-0.8) and 50 nm (Ti-21-1.0) samples is more likely that the thicker ESL is not able to extract electrons as well as the thinner layer, which is seen as a slightly lower PCE [9]. Furthermore, an ESL layer that is too thick can also reduce the light transmittance due to stronger light scattering and greater absorption of photons with energies higher than that of the ESL band gap, and this reduces the photon absorption by the active layer [47,48]. However, when the porous layer becomes thin enough, as in the Ti-21-2.5 sample, more direct shunt pathways are to be expected as the pore channels become shallower perpendicular to the FTO substrate. The large scattering of data points suggests that in some TiO_2_ layers, very few such pathways can be achieved, while others suffer severely from direct shunt pathways. In ultrathin TiO_2_ layers, shunt pathways can also arise from direct contacts between the perovskite and bare patches of FTO due to incomplete surface coverage of the ESL. We previously reported that compact TiO_2_ layers prepared by the dip-coating method need to have a thickness of at least ~30 nm to work optimally in mesoscopic PSCs [23]. However, in the planar configuration, the TiO_2_ layer probably needs to be somewhat thicker (~50 nm) to avoid this kind of pinholes in the devices. 

## 4. Conclusions

We introduced a model system for pinholes in TiO_2_-based ESL layers using block co-polymer-templated thin films with well-defined pore structures. We believe that the investigated system predicts morphological defects, such as narrow pinholes, well. We observed that such pinholes have a very small effect on the overall device performance most likely due to the fact that very few direct pathways of perovskite reach the FTO substrate through the ordered pore system. We saw a slight improvement in *J_SC_* for layers at large porosities as well as a drop in *V_OC_* with increasing porosity. A more interconnected pathway of perovskite forming in the pores when the porosity is high can explain both effects. Larger effects on the device performance are expected if the pore size were to be larger or if the pore structure were to be more open, and we plan to investigate these parameters in a follow-up study. Furthermore, for ultrathin (~20 nm) porous layers, additional direct shunt pathways due to an incomplete ESL surface coverage of the FTO layer further deteriorates the device performance.

## Figures and Tables

**Table 1:** Molar compositions for the dip coating sols used in this study.

	Sol/Sample ^1^	TiCl_4_	EtOH	H_2_O	P2952_BdEO
*Porosity series*					
	Ti-0	1	32.5	8.4	0
	Ti-6	1	36.6	9.5	5.88 × 10^−6^
	Ti-12	1	48.5	12.6	11.8 × 10^−6^
	Ti-21	1	67.9	17.6	21.2 × 10^−6^
*Thickness series*					
	Ti-21-0.8	1	56.2	14.6	21.2 × 10^−6^
	Ti-21-1	1	67.9	17.6	21.2 × 10^−6^
	Ti-21-2.5	1	169	43.7	21.2 × 10^−6^

^1^ The sample names in the porosity series (Ti-*x*) are derived according to the P2952_BdEO/TiCl_4_ molar ratios, where *x* indicates the molar ratio × 10^6^. In the sample names in the thickness series (Ti-21-*y*), the *y* parameter indicates the relative solvent amount (H_2_O + EtOH) in comparison to the original Ti-21 sample.

**Table 2:** Summary of the TiO_2_ film characteristics derived from XRD and XRR data.

Sample	Crystallite Size (nm)	Thickness (nm)	Density (g/cm^3^)	Porosity (%) ^1^
Ti-0	20	75	3.21	0
Ti-6	21	71	2.76	14.0
Ti-12	14	61	2.39	25.5
Ti-21	7	50	1.70	47.0

^1^ The porosity values for the block co-polymer-templated samples are calculated by relating their densities to the non-porous Ti-0 reference sample.

**Table 3:** Mean values and standard deviations of photovoltaic parameters measured in reverse sweep of all type of devices.

Sample	Thickness (nm)	No of Devices	*J_SC_* (mA/cm^2^)	*V_OC_* (V)	FF	PCE (%)
Ti-0	75	13	17.6 ± 0.4	1.11 ± 0.02	0.67 ± 0.03	13.1 ± 0.7
Ti-6	71	18	17.8 ± 0.4	1.10 ± 0.02	0.68 ± 0.03	13.3 ± 0.9
Ti-12	61	13	17.8 ± 0.5	1.10 ± 0.01	0.66 ± 0.03	13.0 ± 0.8
Ti-21	50	19	18.1 ± 0.5	1.09 ± 0.01	0.70 ± 0.03	13.8 ± 0.7
Ti-21-0.8	75	8	17.8 ± 0.3	1.05 ± 0.01	0.69 ± 0.02	12.9 ± 0.4
Ti-21-1.0	50	6	18.1 ± 0.1	1.08 ± 0.01	0.72 ± 0.004	14.1 ± 0.2
Ti-21-2.5	20	7	17.2 ± 2.0	1.01 ± 0.09	0.63 ± 0.11	11.0 ± 2.9

## Supplementary Materials

The following are available online at https://www.mdpi.com/2079-4991/10/1/181/s1, Figure S1: XRR interference patterns of various TiO_2_ thin films, Figure S2: Film thickness and density dependence when varying the solvent amount, Figure S3: MIS-CELIV data, Figure S4: Calculated surface recombination velocities, Figure S5: J-V scans under dark conditions, Figure S6: UV-vis absorption measurements, Figure S7: Steady-state PL measurements, Figure S8: TR-PL measurements, Figure S9: Time-dependent current density measurements, Figure S10: Box charts for photovoltaic parameters of devices where the ESL thickness has been varied.
