# Supplementary material for: Investigation of Well-Defined Pinholes in TiO2 Electron Selective Layers Used in Planar Heterojunction Perovskite Solar Cells
Source: Nanomaterials (Basel). 2020 Jan 20;10(1):181. doi: 10.3390/nano10010181 (PMC7022807; doi:10.3390/nano10010181)
Supplement: Supplementary file 1 [file nanomaterials-10-00181-s001.pdf]

nanomaterials

Supplementary information

Investigation of Well-Defined Pinholes in TiO_2 Electron Selective Layers Used in Planar Heterojunction Perovskite Solar Cells

Muhammad Talha Masood [1,2], Syeda Qudsia [1], Mahboubeh Hadadian [1], Christian Weinberger [1,3], Mathias Nyman [4], Christian Ahläng [4], Staffan Dahlström [4], Maning Liu [5], Paola Vivo [5], Ronald Österbacka [4] and Jan-Henrik Smått [1,*]

1 Laboratory of Molecular Science and Engineering, Åbo Akademi University, Porthansgatan 3-5, 20500 Turku, Finland; mmasood@abo.fi or talha.masood@scme.nust.edu.pk (M.T.M.); squdsia@abo.fi (S.Q.); mhadadia@abo.fi (M.H.); christian.weinberger@upb.de (C.W.)
2 Department of Materials Engineering, School of Chemical & Materials Engineering, National University of Science & Technology (NUST), Sector H-12, 44100 Islamabad, Pakistan
3 Department of Chemistry—Inorganic Functional Materials, Paderborn University, 33098 Paderborn, Germany
4 Physics, Faculty of Science and Engineering, Åbo Akademi University, Porthansgatan 3-5, 20500 Turku, Finland; mathias.nyman@abo.fi (M.N.); cahlang@abo.fi (C.A.); staffan.dahlstrom@abo.fi (S.D.); rosterba@abo.fi (R.Ö.)
5 Faculty of Engineering and Natural Sciences, Tampere University, P.O. Box 541, FI-33014 Tampere, Finland; maning.liu@tuni.fi (M.L.); paola.vivo@tuni.fi (P.V.)
* Correspondence: jsmatt@abo.fi; Tel.: +358-40-5223318

Received: 17 December 2019; Accepted: 16 January 2020; Published: date

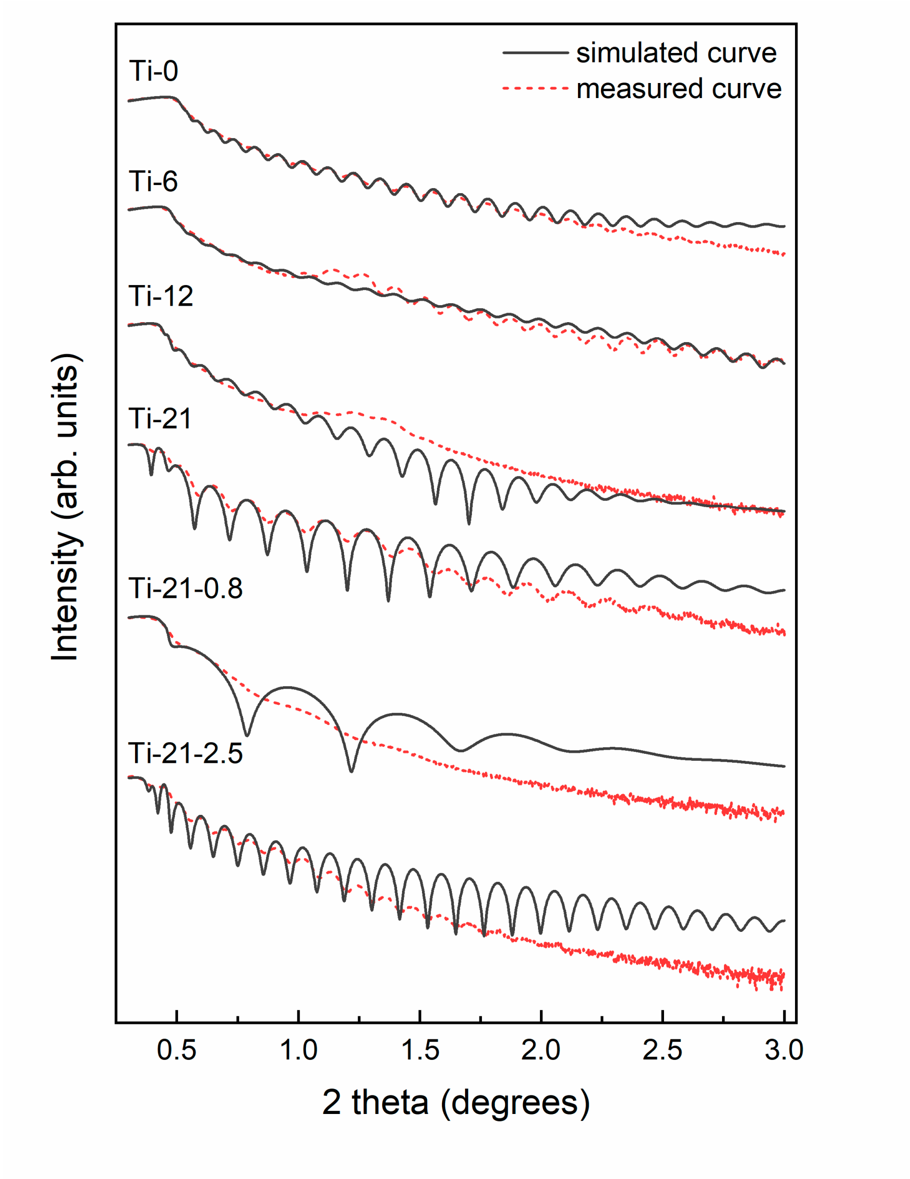

Figure 1. X-ray reflection (XRR) interference patterns of various TiO_2 thin films deposited on glass.

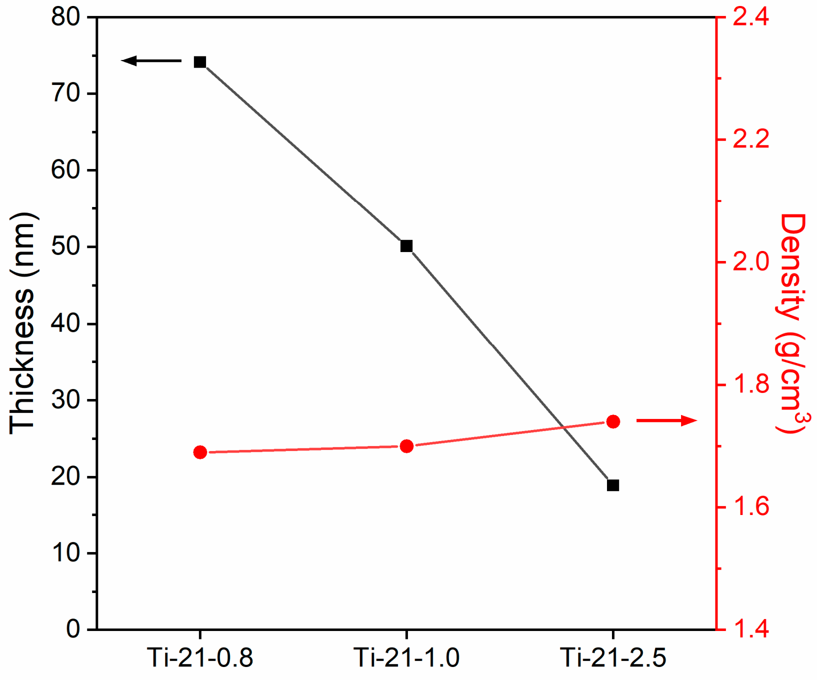

Figure S2. Film thickness and density dependence derived from the XRR data for mesoporous TiO_2 thin films with the highest block co-polymer content when varying the solvent amount.

Description of MIS-CELIV measurements and sample preparation

Surface recombination velocity S_R of holes at the FTO/TiO_2 contact was measured in hole only diodes with device structure FTO/TiO_2/P3HT/Au using the MIS-CELIV technique, which stands for charge extraction by linearly increasing voltage in a metal-insulator-semiconductor structure [1]. In these model devices, holes are injected from the Au top contact through the semiconducting P3HT layer by a steady-state offset voltage V_{off} applied in forward bias. The injected holes are blocked at the hole blocking TiO_2 layer and a charge reservoir is formed at the TiO_2 contact. Subsequently, a linearly increasing voltage pulse is applied in reverse bias of the diode in order to extract the injected hole reservoir. The measured current transient $j(t)$ is given by the sum of the displacement current from the geometric capacitance of the device j_0 and a time dependent linearly increasing extraction current $\Delta j(t)$. The extracted charge Q_{extr} can be determined by integrating the extraction current

$$Q_{extr} = \int_0^{t_{extr}} [\,j(t) - j_0 - J_D\,]dt \qquad (1)$$

where t_{extr} is the time when all injected carriers have been extracted and J_D is the injected dark current density. The surface recombination velocity of holes S_R can be determined from the extracted charge Q_{extr} and the measured steady state dark current $J_D(V_{off})$ at the applied V_{off} according to

$$S_R = \frac{2\varepsilon\varepsilon_0 kT}{qQ_{\mathrm{extr}}^2} J_D(V_{\mathrm{off}}) \qquad (2)$$

where ε is the relative dielectric constant of P3HT (≈ 3), ε_0 is the vacuum permittivity, k is the Boltzmann constant, T is the temperature and q is the elementary charge [1].

A lower S_R value means that the TiO_2 layer is blocking holes more efficiently. Here, the S_R value can therefore be interpreted as a measure of how well the TiO_2 layer prevents the P3HT layer from getting in contact with the FTO substrate, as FTO is not hole blocking. In other words, more intentional pinholes is expected to result in a higher surface recombination velocity. The measured CELIV current transients are shown in Figure S3 and calculated surface recombination velocities are plotted in Figure S4. The S_R values saturate to a constant value in the correct measurement regime at higher V_{off}, clearly larger than V_{bi}, when a large reservoir is accumulated at the TiO_2 layer.

Samples for S_R measurements were made on FTO glass substrates dipcoated with the dip coating solutions listed in Table 1. Spin coating was performed in a nitrogen-filled glove box, using a 40 mg/mL P3HT solution in chlorobenzene, at 1000 rpm for 1 minute and 30 seconds to yield a film thickness of around 300 nm. After spin coating the samples were annealed at 120 °C for around 10 minutes. Finally, 60 nm gold was evaporated on top. Measurements were conducted using a pulse generator (SRS model DG 535) and a function generator (SRS model DS345) for applying the offset voltage and the linearly increasing extraction voltage pulse. An oscilloscope (Tektronix TDS 680B) was used for recording the transient current response. The setup was controlled from a computer using a LabVIEW program.

For the measurements, a voltage rise speed of A = 3 V/ 10 ms was used. Samples were measured at varying applied offset voltage from 0 V to -1.4 V.

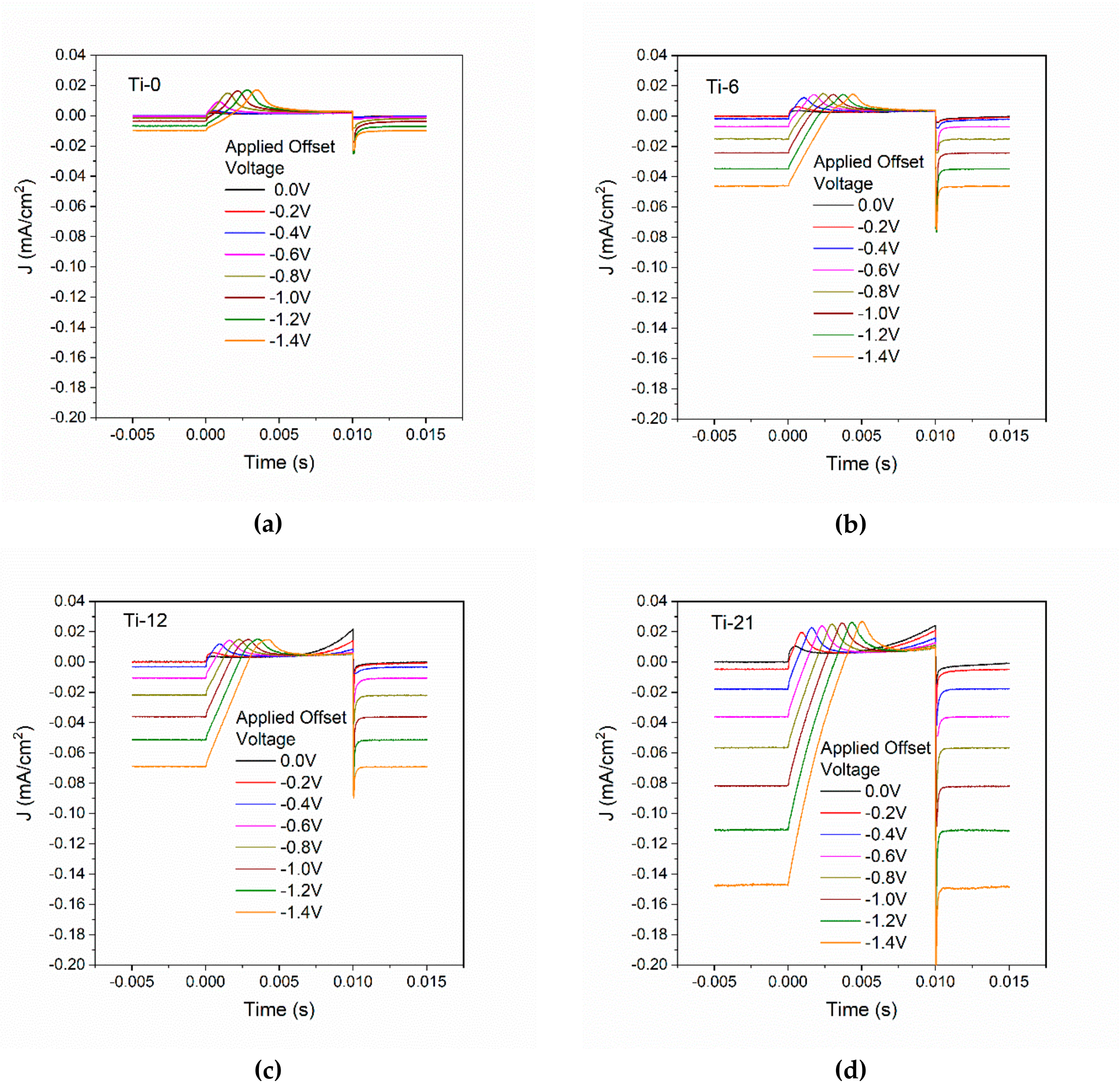

Figure S3. MIS-CELIV data for FTO/TiO_2/P3HT/Au devices with varying porosity in the TiO_2 layer: (**a**) Ti-0, (**b**) Ti-6, (**c**) Ti-12, and (**d**) Ti-21.

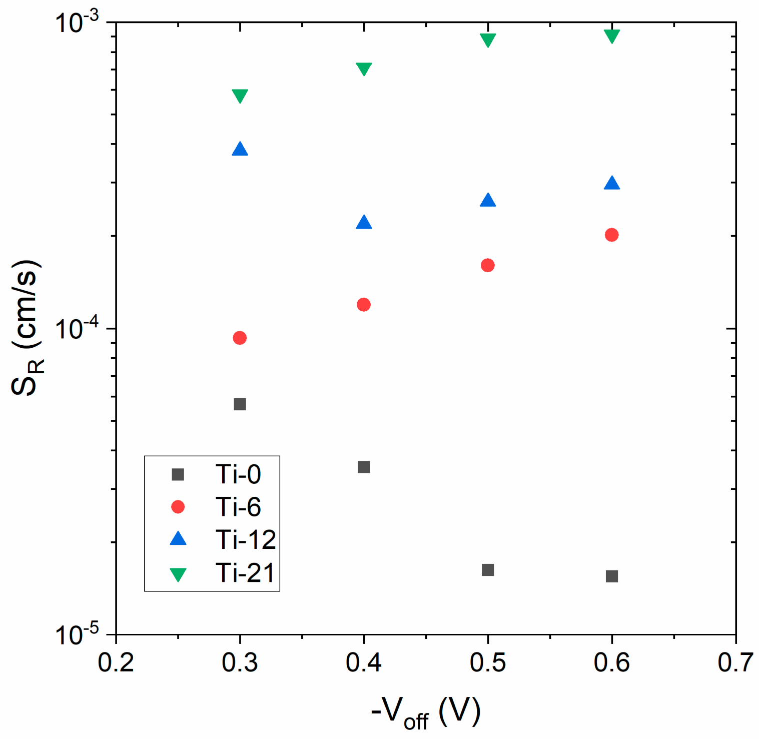

Figure S4. Calculated surface recombination velocities S_R for holes at the TiO_2/P3HT interface from the CELIV data in Figure S3.

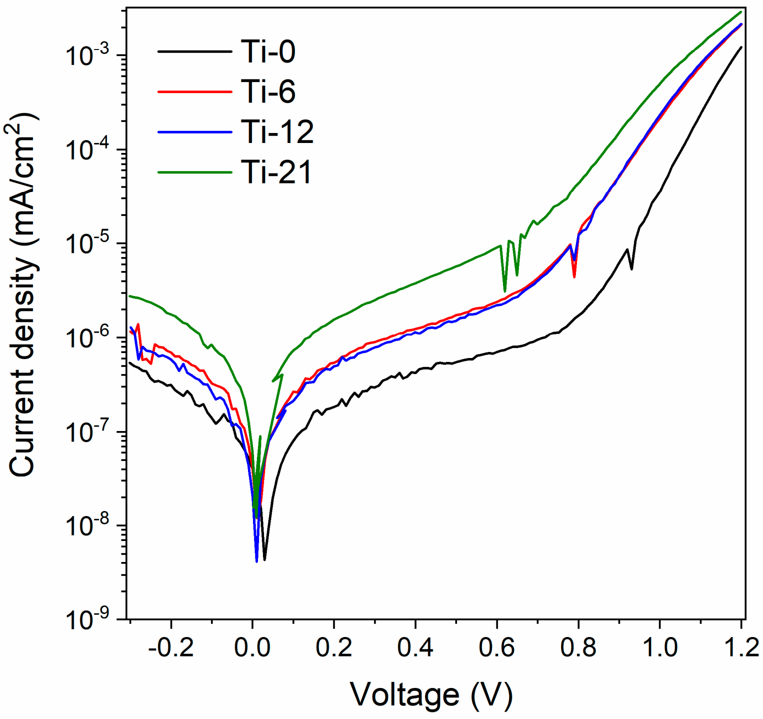

Figure S5. Representative J-V scans measured under dark conditions in forward sweep for devices with increasing porosity in the TiO_2 layer.

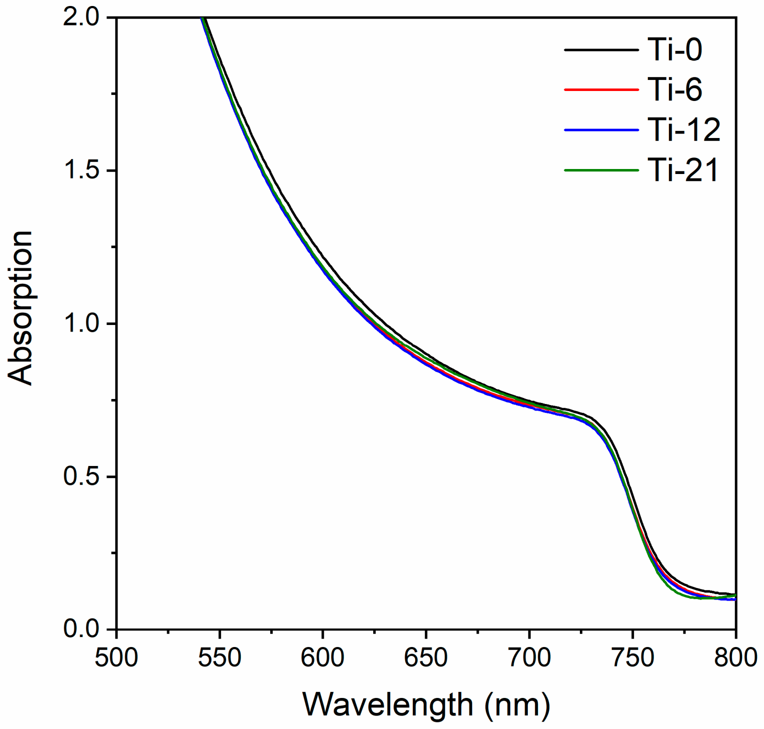

Figure S6. UV-vis absorption measurements performed on TiO_2 thin films with varying porosity deposited on glass substrates.

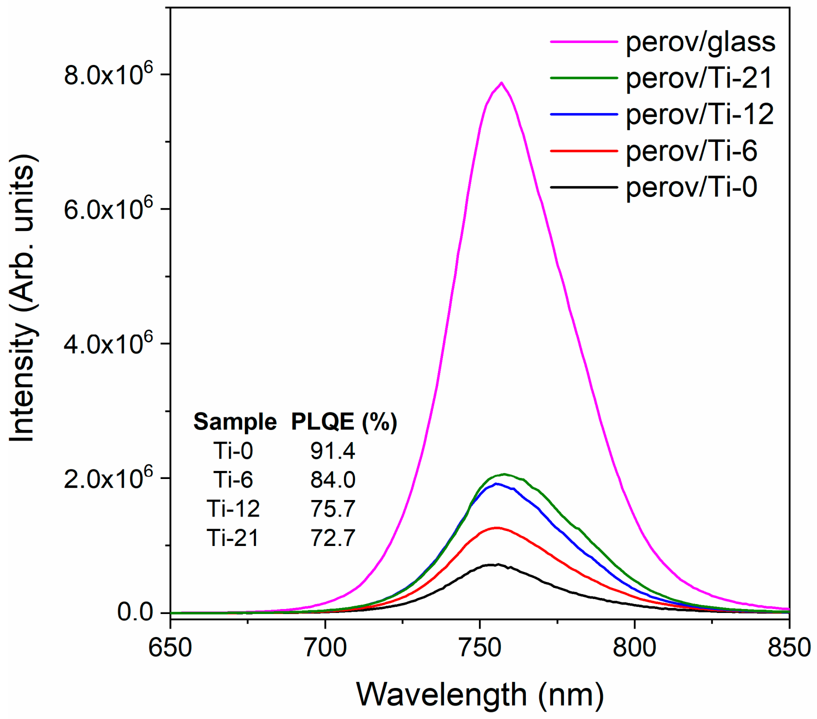

Figure S7. Photoluminescence (PL) spectra of glass/perovskite and glass/TiO_2/perovskite with different porosities of the TiO_2 layer. All samples were excited at 600 nm.

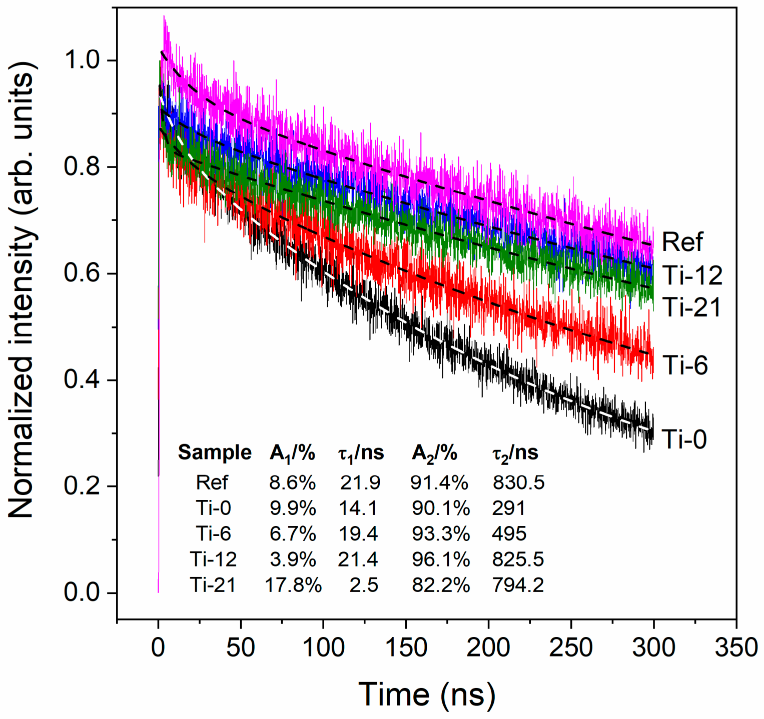

Figure S8. Normalized time-resolved photoluminescence (PL) decays of glass/perovskite reference and glass/TiO_2/perovskite samples with variable porosities of the TiO_2 layer (the dashed lines represent bi-exponential fits of the original data). The table summarizes the bi-exponential fitting results of the PL decays based on the equation: $I(t) = A_1 \cdot e^{(-t/\tau_1)} + A_2 \cdot e^{(-t/\tau_2)}$.

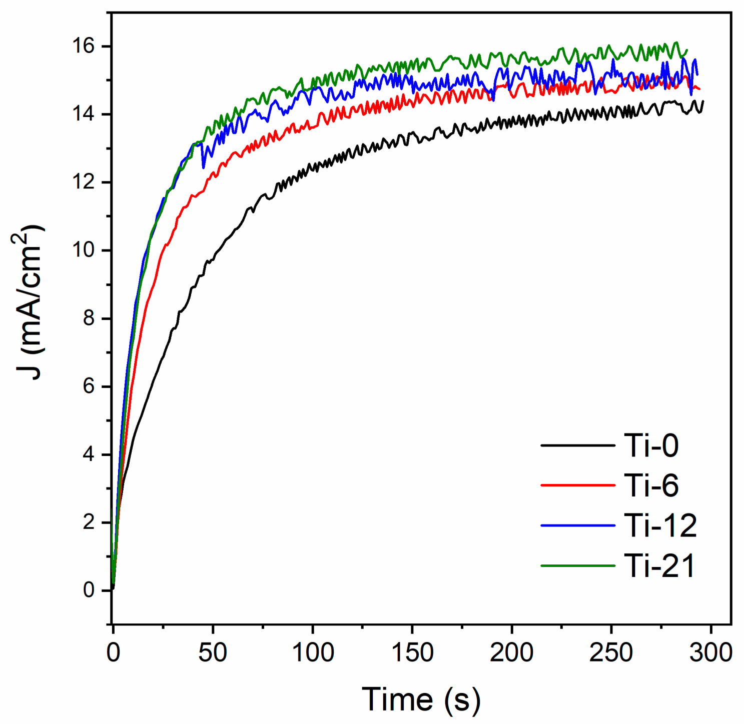

Figure S9. Time-dependent current density measurements performed close to the maximum power point under illumination for devices based on TiO_2 ESLs with varying porosity.

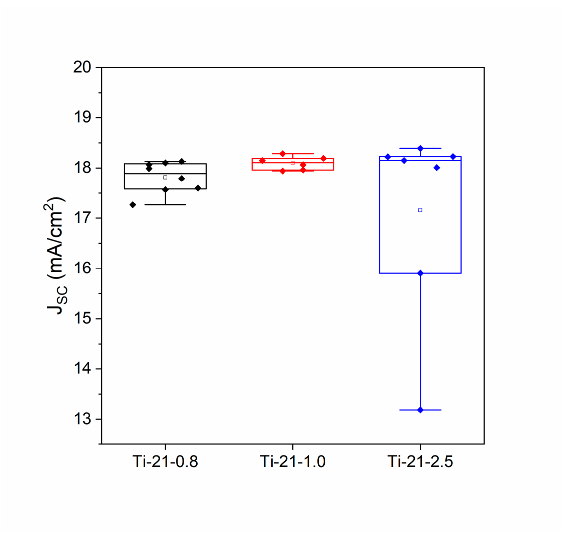

(a)

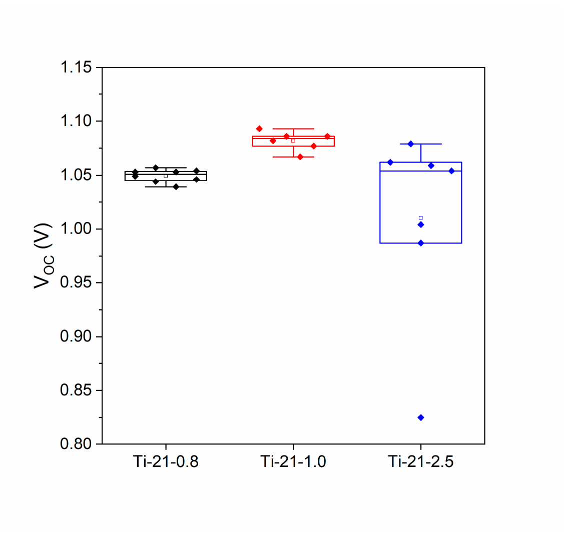

(b)

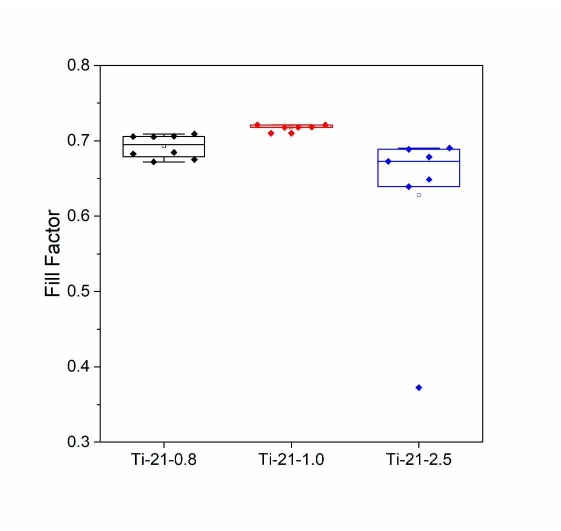

(c)

Figure S10. Box charts for the photovoltaic parameters for devices based on TiO_2 ESLs with the highest porosity but varying thickness: (**a**) J_{SC}, (**b**) V_{OC}, and (**c**) fill factor.

References

[1] Sandberg, O.J.; Sandén, S.; Sundqvist, A.; Smått, J.; Österbacka, R. Determination of Surface Recombination Velocities at Contacts in Organic Semiconductor Devices Using Injected Carrier Reservoirs. *Physical Review Letters* **2017**, *118*, 076601.
